# Use of Residual Lignocellulosic Biomass and Algal Biomass to Produce Biofuels

**DOI:** 10.3390/ijms25158299

**Published:** 2024-07-30

**Authors:** Deborah Terra de Oliveira, Vanessa Albuquerque de Mescouto, Rutiléia de Jesus Paiva, Sara Roberta Ferreira da Silva, Luiz Augusto Barbosa Santos, Gustavo Marques Serra, Luciana Pereira Xavier, Renata Coelho Rodrigues Noronha, Luís Adriano Santos do Nascimento

**Affiliations:** 1 Science and Technology Park-Guamá, Amazon Oil Laboratory, Belém-Pará 66075-750, Brazil; 2 Institute of Biological Sciences, Federal University of Pará, Belém-Pará 47806-421, Brazil

**Keywords:** biofuels, lignocellulosic, waste, microalgae, algae

## Abstract

Efforts are intensifying to identify new biofuel sources in response to the pressing need to mitigate environmental pollutants, such as greenhouse gases, which are key contributors to global warming and various worldwide calamities. Algae and microalgae present themselves as excellent alternatives for solid-gaseous fuel production, given their renewable nature and non-polluting characteristics. However, making biomass production from these organisms economically feasible remains a challenge. This article collates various studies on the use of lignocellulosic waste, transforming it from environmental waste to valuable organic supplements for algae and microalgae cultivation. The focus is on enhancing biomass production and the metabolites derived from these biomasses.

## 1. Introduction

With the global population on the rise, a significant challenge in energy economics is the pursuit of clean energy sources that can supplant fossil fuels like coal and oil, which presently underpin energy production in numerous countries [1]. These fossil fuels are substantial contributors to current climate changes given their release of significant volumes of carbon dioxide (CO_2_) into the atmosphere. Coupled with environmental deforestation, they have been broadly associated with the incidence of heatwaves, hurricanes, tornadoes, acid rain, and desertification observed globally [2]. 

Considerable efforts are being directed towards replacing these fossil fuel sources. The use of plants as raw materials for biofuel production has surfaced as a viable alternative. This includes the utilization of oil-rich crops such as soybean and palm for biodiesel production and high-sugar plants like corn and sugarcane for ethanol synthesis [3,4]. However, given that these crops also form part of the food supply chain, a dilemma arises concerning their use for energy production. This is particularly relevant when considering the potential economic competition that could emerge between markets [5]. 

**Figure 1:**
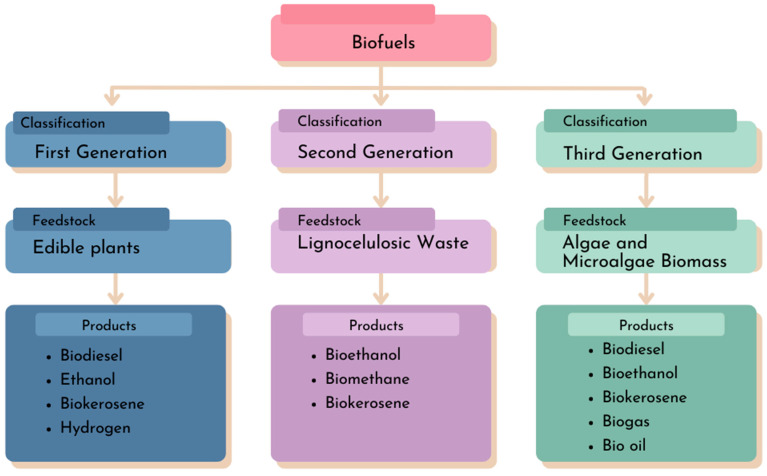
Biofuel generations and bioproducts.

Currently, biofuels, which exist in liquid and gaseous forms, such as biodiesel, ethanol, bio-kerosene, hydrogen, and biomethane, are classified by generations (Figure 1). These generationswere created to address the obstacles related to the use of raw materials [6].

In this context, the first generation of biofuels is distinguished using oil crops such as palm, cotton, and soybean, as well as the employment of corn and sugarcane for ethanol production. The competition between the energy and food markets for these raw materials led to the development of second-generation biofuels, which utilize lignocellulosic residues from non-edible sugar-rich plants. The third generation of biofuels involves the use of microalgae as a raw material for biofuel production, aiming to surmount the challenges faced in second-generation biofuel production. These challenges include the technology needed to decompose highly recalcitrant lignocellulosic material and the use of environmentally detrimental solvents [7,8]. 

Subsequently, algae and microalgae, as photosynthetic organisms, have surfaced as a solution to the challenges encountered in the first and second generations of biofuels. This is due to their versatility in generating different metabolites and, consequently, a variety of bioproducts [9]. These organisms are simpler to cultivate as they require fewer resources for their growth, such as water, light, carbon dioxide (CO_2_), and nutrients like nitrogen and phosphorus. Furthermore, under conditions of cellular stress, they tend to accumulate metabolites as energy reserves, including specific lipids [10] and pigments [6,11].

Some of the metabolites generated by algae and microalgae carry significant economic value. They are utilized in the food industry for the production of dietary supplements and in the cosmetics market as ingredients with antioxidant activity, thickeners, and pigments. They also hold relevance in the pharmaceutical market for bioactive compounds with antiviral activity and vitamins, as demonstrated in Table 1 [2,12,13].

Moreover, to extract the various metabolites that algae and microalgae can produce, specific cellular stress techniques are utilized to obtain commercially valuable primary and secondary metabolites. For example, in the study conducted by Asimakopoulou et al. [18], the cultivation of the microalga *Crypthecodinium cohnii* was supplemented with carbon sources derived from agro-industrial waste. This was performed with the aim of producing omega fatty acids, which are sold as dietary supplements for animals and humans. The study successfully achieved a yield of approximately 32.2% docosahexaenoic acid (DHA), also known as an omega-3 fatty acid.

Microalgae can also accumulate pigments as secondary metabolites, which are marketed as colorants in the food and cosmetics industries. This was observed in experiments conducted by Vyas et al. [11], where cultures of *Chlorella sorokiniana* were supplemented with spruce hydrolysate. This resulted in the accumulation of 6.4 mg.g_CDW_^−1^ (milligram per gram of cell dry weight), which has anti-inflammatory and antioxidant bioactivities. Similarly, the study by Norzagaray-Valenzuela et al. [19] demonstrated antioxidant activity in protein extracts from *Dunaliella tertiolecta* DUT2, *Tetraselmis suecica* TES2, and *Nannochloropsis* sp. NNX1. This suggests that the observed activity may be due to the presence of phenolic compounds, carotenoids, polysaccharides, and other bioactive compounds in the biomass of these strains.

Beyond these bioproducts, algae and microalgae serve as a potential source for clean energy production in the synthesis of liquid-gaseous biocompounds thanks to the high-energy products present in their biomass composition. As reported by Rempel et al. [20], carbohydrates derived from the cultivation of *Spirulina platensis* achieved an efficiency of over 80% in their conversion to bioethanol. Additionally, they generated biomethane with a high energy potential of 13.9 kJ.gbiomass^−1^ (kJ per gram of biomass) from the biomass residues used in bioethanol production. In a similar vein, Tsai et al. [21] conducted a study with *Synechococcus elongatus* PCC7942, where its biomass was used for biobutanol and hydrogen production. The production capacity ranged from 0.3 to 4.3 g.L^−1^ of biobutanol and 2502 to 7911 mL.L^−1^ of hydrogen.

As a raw material for energy production, fatty acids, wich constitute the lipids produced by algae and microalgae can be used to produce high-quality biodiesel, as observed by Chen et al. [22]; they evaluated the cultivation of *Chlorella protothecoides* supplemented with sugarcane bagasse hydrolysate as a carbon source and obtained over 16.8% of fatty acids suitable for biodiesel synthesis. However, despite the various advantages, obtaining large volumes of biomass from algae and microalgae remains one of the observed obstacles that are being investigated for these organisms to serve as substitutes for raw materials in biocompound production. As seen in the studies above, supplementation with carbon sources using residual lignocellulosic material is an alternative method to increase biomass production and, consequently, the production of metabolites used in biocompound synthesis.

The study conducted by Spennati et al. [23] assessed the use of winery wastewater in the cultivation of *Chlorella vulgaris* and *Arthrospira platensis*, resulting in approximately 5.5 gDW.L^−1^ of biomass and a reduction in water pollutants such as polyphenols by approximately 50–60%. Miazek et al. [24] utilized beech wood (*Fagus sylvatica*) for the supplementation of *Chlorella sorokiniana* and observed an increase in biomass production (50%), fatty acids (from 3% to 6%), and pigments (7.8 ± 0.9 mg.L^−1^.d^−1^).

Therefore, the purpose of this article is to discuss the utilization of residual lignocellulosic biomass as a carbon source for the cultivation of algae and microalgae, as well as the enhancement of metabolite production with high biotechnological value used in the production of solid-gaseous biofuels.

## 2. Research Methodology

The search strategy employed in this article adhered to the PRISMA methodology [25]. The document search related to the article’s topic was conducted using the Scopus, Web of Science, and PubMed databases, with the searched topics presented in Table 2. The eligibility criteria for the documents’ inclusion in this article were confined to research articles and reviews published between 2018 and 2023. During a second screening, documents unrelated to the topic and duplicate articles, identified by title, abstract, and keywords, were excluded.

**Figure 2:**
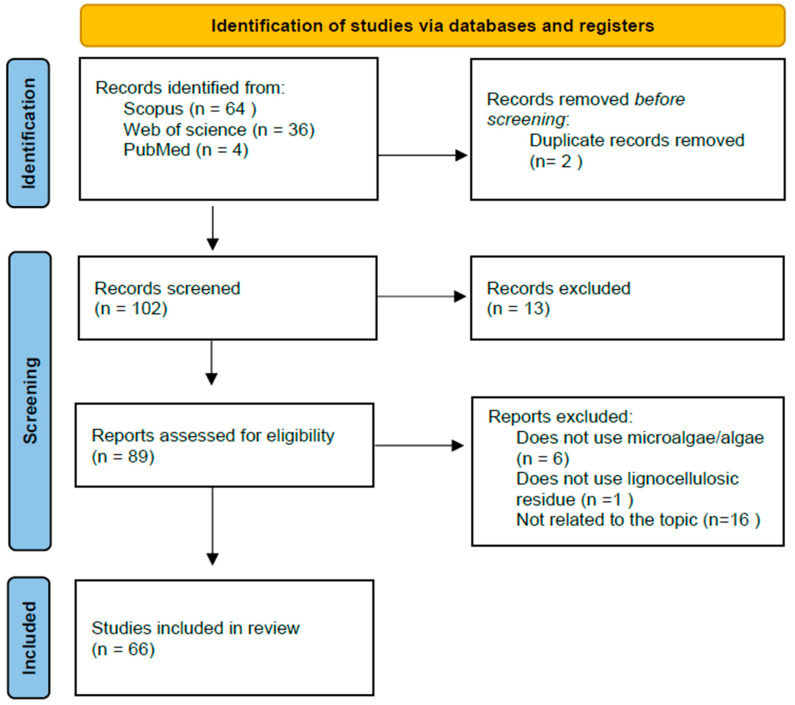
PRISMA flow diagram indicating the search strategy.

The final screening was carried out by three evaluators using an automated tool. Articles that did not involve the use of microalgae for biofuel production or the use of lignocellulosic biomass for the enrichment of microalgal biomass or for biofuel production with microalgae were excluded. The methodology flowchart is shown in Figure 2.

## 3. Results

### 3.1. Algal and Microalgal Biomass

Algae represent a polyphyletic group of photosynthetic organisms, characterized by a broad diversity of forms, functions, and adaptation strategies. This group encompasses both eukaryotic and prokaryotic algae (cyanobacteria), often studied in conjunction due to their analogous photoautotrophic metabolism. Algae display an extensive range of morphologies, from single-celled organisms to cell aggregates, colonies, and filaments. They exist in diverse forms, spanning unicellular microalgae such as *Chlorella* spp. to multicellular macroalgae like *Ulva* spp. [26,27].

**Figure 3:**
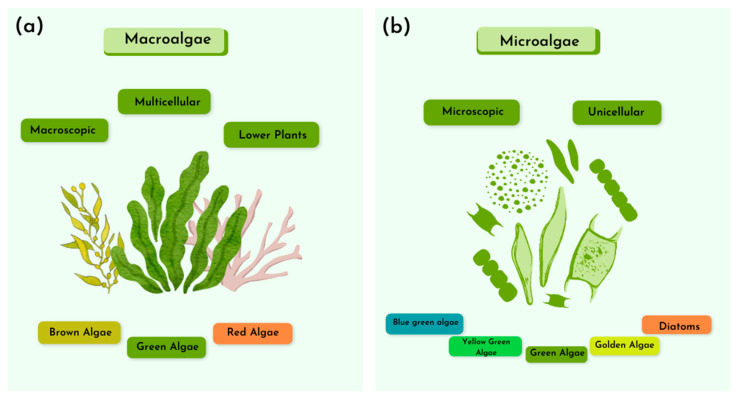
Main characteristics of macroalgae (**a**) and microalgae (**b**).

Macroalgae are primarily found in marine environments and belong to the group of non-vascular plants. Unlike higher plants, they lack specialized structures and reproductive methods. Macroalgae can be observed with the naked eye due to their larger size. Because of their size, macroalgae are considered potential sources of biomass for bioenergy production, particularly for bioethanol and biogas [26]. The composition of macroalgae determines their energy pathway. Macroalgae that are rich in lipids can be used for biodiesel production. Furthermore, macroalgae can be classified into three main groups based on their pigment content: red algae (*Rhodophyta*), brown algae (*Ochrophyta*), and green algae (*Chlorophyta*) (Figure 3).

Microalgae are microscopic organisms that play a crucial role in photosynthesis and the production of organic matter in marine ecosystems. They are also important for energy conversion and the assessment of water bodies [28]. Additionally, microalgae are a promising source of valuable bioproducts with a wide range of industrial applications, including the treatment of industrial and domestic waste [15,29]. To fully understand these properties of microalgae, it is necessary to study the microorganism, including its structural characteristics, nutrient requirements, and the bioactive compounds it produces. Compared to terrestrial plants, microalgae are sustainable due to their rapid growth, ease of cultivation, and the fact that they do not compete for arable land.

These unicellular microorganisms encompass over 50,000 classified species. Some notable genera include *Chlamydomonas, Arthrospira, Aphanizomenon, Chlorella, Stigeoclonium,* and *Spirulina* [15,30,31,32]. The cell wall composition of microalgae varies among species. Some microalgae cells have strong components in their cell wall, such as alginate. This macromolecule provides resistance in the outer layer of certain species. Typically, the cell walls of the *Chlorella* genus are composed of glycoprotein structures and carbohydrates, including glucose, xylose, rhamnose, and galactose [33].

The various types of cell coatings are related to the needs of each microalgae and their habitat, including interactions with chemical substances, communication, cell-to-cell connection (as they can live in filamentous forms), reproduction, maintenance of cell shape, and substrate fixation [2,34]. In addition to their characteristics regarding cell coatings and primary metabolites (e.g., proteins, carbohydrates, fatty acids, and lipids), the main focus of studies and industrial application exploration is related to the presence of high-value secondary metabolites [6].

The average lipid content of microalgae varies between 1 and 70% (w.w^−1^) [6] and depends on the cell species, life cycle, and nutritional and environmental requirements of microalgae. In some species, the lipid content can reach up to 80% of the dry cell weight. Well-known PUFA producers include *Crypthecodinium, Schizochytrium*, and *Ulkenia* spp. The influence of glucose concentration not only increases biomass and lipid accumulation but also affects the PUFA production profile in *Schizochytrium* spp. [35].

Residual microalgae biomass is not only interesting due to the quantity of PUFAs that can be obtained but also due to its high protein and peptide content. Therefore, it has the potential to serve as an alternative source of proteins that could replace conventional food proteins in developing countries [11].

In the study by Vyas et al. [11], the potential of the microalga *Chlorella sorokiniana* as an abundant and important source of protein production was evaluated. In the study, proteins accounted for 416.6 ± 29.7 mg.g^−1^ of cell dry weight (cdw) in photoautotrophic cultures of *C. sorokiniana*, whereas under heterotrophic conditions, it yielded 162.7 ± 4.9 mg.g^−1^ wt. It is worth noting that the profile and fractions of soluble proteins decreased as the growth shifted from photoautotrophic to heterotrophic conditions. These findings suggest that cellular energy was redirected towards lipid metabolism and the production of other primary metabolites essential for cell survival.

Depending on the microalgal strain and nutritional conditions, the chlorophyll content of a cell can vary from 0.1 to 1% wt [6]. *Chlorella* species are the main producers of chlorophyll [36]. In addition to its previously described photosynthetic role, chlorophyll has been shown to exhibit antioxidant protection capability [19]. In cyanobacteria and red algae, the main class of light-capturing pigments is the pigment–protein complexes called phycobiliproteins. These hydrophilic pigments absorb light in the visible spectrum between 500 and 650 nm and assist chlorophylls in maximizing light capture. They are divided into four main classes: phycocyanins, phycoerythrins, phycocyanin-phycoerythrin, and allophycocyanin [6].

### 3.2. Lignocellulose Waste

#### Types of Lignocellulosic Waste and Applications

Due to an increase in energy consumption influenced by population growth and rapid urbanization, the use of fossil fuels as an energy source has raised various concerns, such as environmental damage through increased greenhouse gas emissions, among others. Consequently, the search for sustainable sources to reduce dependence on fossil fuels has become one of the main global issues addressed, with biorefineries emerging as sustainable alternatives to address these challenges [37]. One sustainable possibility for energy production is the utilization of biomass, which can be obtained from organic waste, food waste, and agricultural residues, among others. Lignocellulosic biomass is the most abundant source of biomass for energy purposes [38].

Lignocellulose can be found in various raw materials, such as hardwood, softwood, grasses, agricultural residues, forestry residues, and food industry waste [18,39]. The effective implementation of an integrated biorefinery depends on utilizing all components of the raw material to maximize and diversify production [40].

Due to their low cost and renewable nature, lignocellulosic biomasses have emerged as a viable alternative for economic development as they can contribute to the production of different products through autonomous processes and their reuse. Traditionally, they have been used in combustion processes for energy production. However, recent research has focused on the production of biofuels such as bioethanol and biomethane, as well as biofertilizers and various high-value bioactive compounds through biorefinery processes [37].

The main components of the compact and recalcitrant structure of lignocellulosic biomass are cellulose, hemicellulose, and lignin, with the content of each constituent varying depending on the plant species [41,42]. The complex structure of this matrix hinders the isolation and recovery of derivatives from each component, such as sugars present in cellulose and hemicellulose and fertilizers and polymers derived from lignin breakdown. Sequential pretreatments are often necessary to make these products accessible, including chemical, physical, biological, or even consortium pretreatments [40].

**Figure 4:**
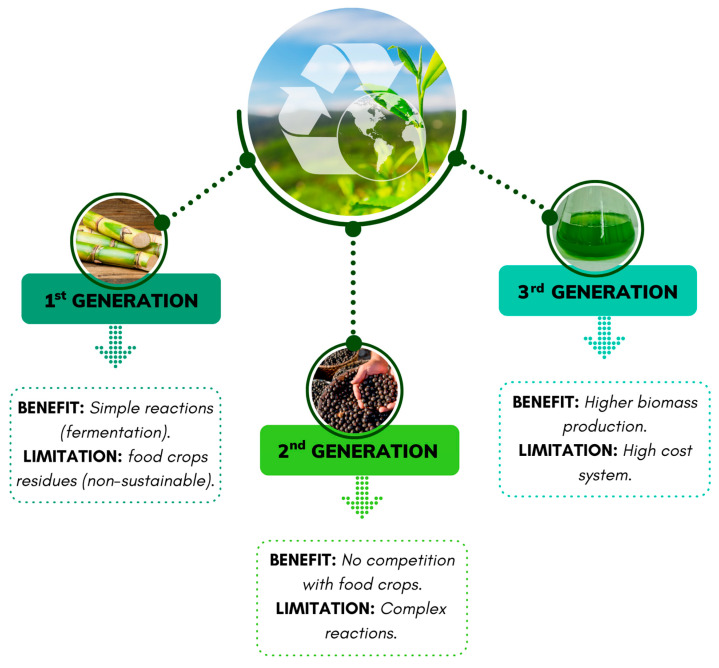
Benefits and limitations of agro-waste generations.

Agro-industrial residues are excellent sources of animal feed and supplementation of microbial cultures in fermentation processes due to their rich nutritional composition. Based on their composition and plant source, agro-industrial residues are classified into three groups: first-, second-, and third-generation (Figure 4). First-generation agro-industrial residues are rich in starch, oil, and sugars, commonly obtained from food sources such as corn and sugarcane, and are used for biofuel production. However, biofuel production from these residues is categorized as unsustainable. Second-generation feedstocks consist of non-edible plants and lignocellulosic biomass, such as jatropha and millet, among others. These sources are often used for biofuel production or the production of compounds with added commercial value. Finally, the third generation consists of photosynthetic microorganisms, such as microalgae and cyanobacteria, which are rich sources of lipids [37].

**Figure 5:**
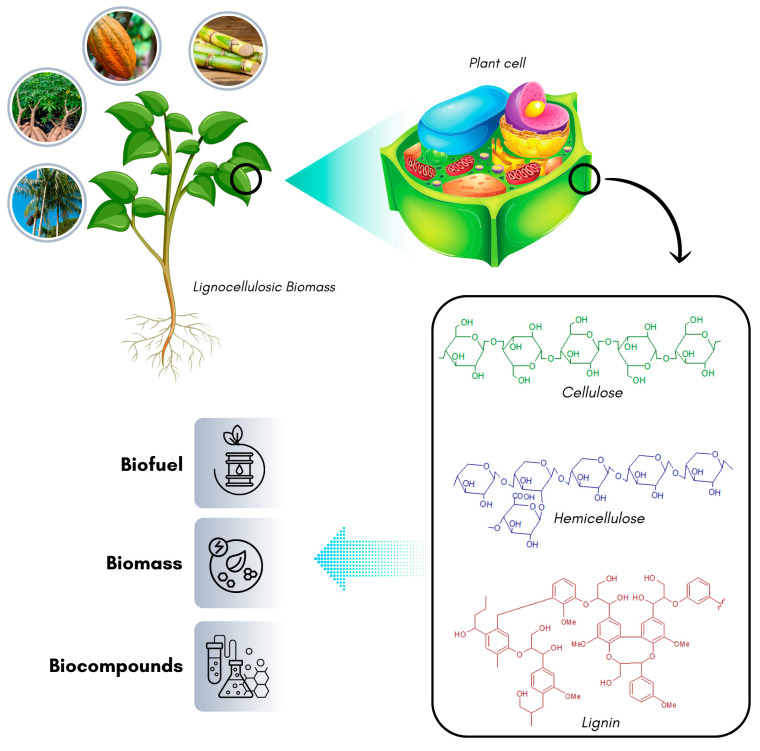
Structure of lignocellulosic residues used to obtain products with added value.

One way to utilize residual lignocellulosic biomass is by obtaining its composition fractions and transforming them into high-value-added products. The sugars present in the biomass have been investigated as carbon sources for supplementing microbial culture media. These sugars assist in increasing the biomass production of these microorganisms, which is considered an essential process that directly influences the cost of the fermentation process. Thus, the utilization of lignocellulosic residues makes the process economically feasible due to the low cost of lignocellulosic sources [43] (Figure 5).

The study conducted by Asimakopoulou et al. [18] utilized lignocellulosic waste, focused on the integrated valorization of its constituent sugars, in which, they obtained wheat straw hydrolysate and used it as a supplement for the cultivation of the *microalga Crypthecodinium cohnii* to produce polyunsaturated omega-3 fatty acids (PUFAs), specifically docosahexaenoic acid (DHA). The total fatty acid content obtained in the dry biomass was approximately 70%, of which 32% corresponded to DHA. Using the same system for the hydrolysis of lignocellulosic biomass, Asimakopoulou et al. obtained 31.3 g.L^−1^ and 11.9 mg.L^−1^ of polyhydroxyalkanoates and astaxanthin, respectively, by cultivating *Paracoccus* sp. LL1 with corncob hydrolysate [44].

Therefore, the current industrial processes aim to achieve sustainability. The concept of sustainability encompasses various aspects, such as social, economic, and environmental factors, with an emphasis on minimizing waste production, among other factors. To develop a sustainable process, it is necessary to reduce the volume of generated waste or even utilize it in different processes by adding value [43].

### 3.3. Structural Components of Lignocellulosic Residues

#### 3.3.1. Cellulose

Cellulose (C_6_H_10_O_5_) is the main constituent of lignocellulosic substrates (approximately 35–55%). It is a linear homopolymer composed of 4000 to 8000 glucose residue units linked by β (1–4) glycosidic bonds. The cellulose chains are naturally polymerized through Van der Waals forces and hydrogen bonds, forming cellulose fibers that can exist in highly crystalline forms, limiting the accessibility to enzymes or chemical catalysts, as well as in amorphous forms [37,39].

Cellulose is highly important as it provides strength and performs various functions, including shaping, division, and differentiation in plants. Cellulose fibers are arranged in an amorphous matrix of lignin, pectin, and hemicellulose. Other subunits, apart from cellulose, are embedded within the cellulose microfibrils in cell walls. The fibrils of this biopolymer are surrounded by a lignocellulosic matrix, making them highly resistant to enzymatic hydrolysis [45].

Cellulose is the most common organic compound on Earth found widely in nature, mainly in plants, fungi, and algae. It is a high-molecular-weight homopolysaccharide that decomposes at 240–350 °C to produce anhydrocellulose and levoglucosan [45,46].

The primary industrial source of cellulose is wood pulp (85–88%), but it can also be derived from cotton linters (12–15%). However, several factors influence the cellulose content, such as the age and nature of the plants [45].

#### 3.3.2. Hemicellulose

Hemicellulose is an amorphous polymer and a heterogeneous group of short-chain heteropolysaccharides (DP~200) composed of monosaccharides. These sugar units can be pentoses (L-arabinose and D-xylose), hexoses (D-glucose, D-mannose, and D-galactose), and other traces of acetic acid, ferulic acid, p-coumaric acid, and glucuronic acid. In addition to these main components, smaller amounts of sugars, including L-rhamnose and L-fucose, have also been reported. The most important monomers are xylose, mannose, arabinose, and glucose. Xylose is an important carbohydrate that contributes 80% of the total sugar present in the hemicellulosic fraction. Therefore, its complete hydrolysis may require different enzymes and constitutes about 20–40% of lignocellulosic substrates [39,45,47].

After cellulose, hemicellulose is one of the main components of biomass, along with cellulose and lignin, in the cell walls of plants. Its quantity can also vary depending on the age and type of raw material [45]. The presence of hemicellulose increases the overall stability of lignocellulose [47]. Its decomposition (200–260 °C) produces more volatile compounds, less tar, and less char than cellulose [46]. However, the degradation of both components (cellulose and hemicellulose) produces furfural, 5-hydroxymethylfurfural (HMF), and weak organic acids.

These compounds interfere with cell replication and enzymatic activity related to energy metabolism (glycolytic and TCA pathways), but their inhibitory effect is generally observed at concentrations higher than 1.0 g.L^−1^ [39]. Due to the presence of branched groups in the side chain, this fraction is non-crystalline and more open. The open structure of this fraction is one of the main reasons that makes it more hygroscopic than cellulose and attracts more water molecules. In addition, its high amorphous nature makes it more suitable than cellulose for hydrolysis. Hemicellulose is considered useful in many fields, such as chemistry, food, medicine, and energy. However, it is a more promising material for industrial products [45].

#### 3.3.3. Lignin

Lignin is a heteropolymer made of phenolic precursors, resulting in high resistance to biodegradation, and it accounts for 5 to 30% of the composition of lignocellulosic biomass [39]. As demonstrated by Shrivastava and Sharma [47], its self-aggregated 3D structure, often referred to as “self-assembled supramolecular chaos”, make it chemically stable and complex, assuming difficulties for its utilization in its natural form. It is the second most abundant biopolymer, after cellulose, representing nearly 30% of Earth’s organic carbon. The random arrangement of the basic building blocks, the monolignol units, and a complex matrix of aliphatic and phenolic substances contribute to its recalcitrant nature [45]. Lignin decomposes at 280–500 °C, producing inhibitory compounds, such as phenolic compounds. Its decomposition yields more charcoal than cellulose [45]. Microorganisms or enzymes can be used to selectively degrade lignin from hemicellulose in lignocellulosic substrates [39].

In general, the content of cellulose, hemicellulose, and lignin in lignocellulosic materials falls within the range of 35–55%, 20–40%, and 5–30%, respectively. However, it is known that these levels vary depending on the lignocellulosic substrate (Table 3).

### 3.4. Pretreatment of Lignocellulosic Biomass

Lignocellulosic residues are recyclable sources of nutrients, such as carbohydrates, for the cultivation of photosynthetic microorganisms. The main lignocellulosic components present in these residues (cellulose, hemicellulose, and lignin) are strongly interconnected by chemical and hydrogen bonds, forming a dense and stable structure. In order to overcome this recalcitrant structure, pretreatment steps (physical, chemical, biological, mechanical, enzymatic, and others), or a combination of these processes are required before biomass utilization, to make sugars more accessible to microorganisms. These pretreatment methods play an important role in assessing the costs of these processes, mainly through the delignification or solubilization of the cellulose and hemicellulose fractions to make them more available for hydrolysis [10,39,52].

There are several types of pretreatment available for lignocellulosic biomass, each method being divided into its specialized pretreatment and directed according to the requirements of the respective extraction compound to be extracted. After this stage, the pretreated residues undergo fermentation or digestion [10]. Some types of pretreatment specializations covered in this review are listed below.

#### 3.4.1. Physical Pretreatments

Physical pretreatment is a method characterized by obtaining fine and amorphous structures, increasing pore size, and altering the structure of lignocellulosic materials to facilitate the separation of their components. However, these methods typically require high energy input, which affects processing costs. The main examples of this procedure include mechanical methods, subdivided into microwave and ultrasound treatments, as well as thermal methods. The mechanical process involves reducing the particle size of the raw material to release the organic fraction into the liquid phase, while the thermal method utilizes heat, with temperature being the main factor in breaking down or decomposing the rigid structure of lignocellulosic residues. Thermal pretreatment can be classified as a low temperature (<100 °C) or a high temperature (>100 °C) [10,39]. 

#### 3.4.2. Chemical Pretreatments

The chemical process can disaggregate the material with low costs, reduced energy consumption, and increased efficiency by employing a hybrid combination of chemical and mechanical pretreatments. This pretreatment method is divided into subclasses: acid pretreatment, which involves the application of strong and weak acids such as hydrochloric acid (HCl), sulfuric acid (H_2_SO_4_), and citric acid (C_6_H_8_O_6_), alkaline pretreatment, which is similar to the acid approach but uses strong and weak alkaline chemicals, such as sodium hydroxide (NaOH), potassium hydroxide (KOH), and calcium hydroxide (Ca(OH)_2_), and *organosolv* pretreatment, which utilizes an organic solvent for the delignification of the lignocellulosic material, leading to the solubilization of hemicellulose in the aqueous phase and removal of lignin in the organic phase. This approach positively contributes to the accessibility of cellulose in the hydrolysis step [10,13,53].

#### 3.4.3. Biological Pretreatments

Biological pretreatment is a sustainable method as it does not release pollutants into the environment. It is subdivided into enzymatic pretreatment and fungal pretreatment. These methods do not require significant energy demands or investments; however, they require a longer processing time. The biological pretreatment of food waste, for example, is favorable for the cultivation of photosynthetic microorganisms such as microalgae due to the presence of various soluble macronutrients. The carbon present in the waste can be used for the growth of these microorganisms while the remaining portion can be processed later for bioenergy, adding value and providing appropriate disposals for all fractions of the waste, thereby reducing environmental impacts [10].

### 3.5. Enrichment of Algal and Microalgal Biomass with Lignocellulosic Waste to Produce Biofuels

The accumulation of metabolites such as lipids, carbohydrates, and proteins in the biomass of microalgae and algae is fundamental for the production of economically interesting biofuels. The production of these metabolites can be directed through the use of lignocellulosic waste as a substrate. It is known that microalgae are capable of accumulating 20–50% of lipids in their dry weight from the stimulus generated in their cultivation. Similarly, the production of proteins and carbohydrates can also be induced from the supplementation of the culture medium [1,4,16,37].

One of the major challenges in cultivating microalgae is to increase biomass production in a way that makes it competitive in the market. Thus, the use of cellulose obtained from lignocellulosic waste has proven to be a strong alternative for the cultivation of microalgae, acting as a carbon source for cells through the provision of sugars, resulting in the reduction of microalgal biomass production costs. In addition, this approach offers environmental and economic benefits to the local population, with the purpose of providing a use for waste that would be discarded [3,5,12].

Various studies are being conducted to take advantage of residual lignocellulosic biomass that can enhance the growth of microalgae. For example, Tsolcha et al. [54] used winery waste to increase the biomass of the cyanobacteria *Leptolynbgya* spp., achieving 32.2 g.L^−1^ of bioethanol. Similarly, Liu et al. [55] used cassava waste to increase the production of *Chlorella pyrenoidosa* biomass, achieving 18.3 g.L^−1^ and producing 42% of lipids that can be used in biodiesel production.

Nguyen et al. [35] evaluated the influence of lignocellulosic waste on biomass and lipid production and observed that the cultivation of *Schizochytrium* spp. in sugarcane bagasse waste was able to produce 10.45 g.L^−1^ of biomass, with 45% of lipids in dry biomass.

In general, the supplementation of microalgae and algae cultures with lignocellulosic waste presents itself as a promising strategy for the production of biofuels. This approach integrates environmental benefits with social and economic gains, ensuring a sustainable and better energy future for future generations.

#### 3.5.1. Biodiesel

Algae and microalgae biodiesel have emerged as a great alternative to the fossil fuel sources currently used. This is because they come from a renewable and cleaner source and their biomass is rich in saturated and monounsaturated fatty acids, which are ideal for the synthesis of high-quality biodiesel. Biodiesel is a biofuel obtained through the transesterification reaction, which involves the chemical breakdown of triglyceride structures. When triglycerides encounter alcohol and a catalyst, they react and form esters and glycerol as products [56].

Considering that biomass production is currently the greatest challenge for the utilization of algal biomass, supplementation with lignocellulosic hydrolysates has been an option to increase metabolite production in algae and microalgae cultivation. In this regard, Sakthi Vignesh et al. [57] evaluated the effect of supplementation with lignocellulosic waste hydrolysate in the cultivation of microalgae *Coelastrella* sp. M-60 and *Dictyococcus* sp. VSKA18 for biodiesel production. They obtained a lipid content of 44% and 52%, respectively, with approximately 90% composed of saturated fatty acids for the *Dictyococcus sp*. VSKA18 strain and around 28% of saturated fatty acids in the biomass of *Coelastrella* sp. M-60, which met the international quality parameters for biodiesel. 

Among the wide range of biodiversity of lignocellulosic residues, sugarcane straw has been extensively studied due to its large-scale use in first-generation ethanol production and its commercialization in the food market to produce molasses, sugars, and sweeteners. In this regard, Nguyen et al. [35] investigated the influence of lignocellulosic hydrolysates on microalgae cultivation using sugarcane bagasse as a carbon source for *Schizochytrium* spp. They obtained a lipid content of 45% as a result of supplementation in the cultivation. The lipid profile of the obtained oil showed an optimal balance between saturated fatty acids (SFAs) and monounsaturated fatty acids (MUFAs), with high levels of palmitic acid (C16:0) and oleic acid (C18:1), and low production of polyunsaturated fatty acids (PUFAs), ensuring the production of biodiesel with excellent quality parameters.

Mu et al. [58] also tested sugarcane hydrolysate on a strain of *Chlorella protothecoides* and found that the hydrolysate was much more efficient than glucose in increasing biomass and lipid content, reaching a lipid content of 34% and a lipid production of 439 mg.L^−1^.d^−1^. Liu et al. [55] evaluated the effect of supplementation with cassava bagasse hydrolysate in *Chlorella pyrenoidosa* cultures, obtaining lipid contents of 38%, 42%, and 43% as the sugar concentration in the medium increased. When analyzing the lipid profile, palmitic acid (C16:0) and oleic acid (C18:1) were found in higher quantities, similar to what is found in oil-producing plants.

Miazek et al. [24] investigated lignocellulosic residues to synthesize beech wood (*Fagus sylvatica*) hydrolysate and supplemented the culture of *Chlorella sorokiniana*. They observed an increase in lipid production with a fatty acid content of 5.2% of dry biomass and a fatty acid production rate of 12.6 mg^−1^.d^−1^. These results demonstrated that the hydrolysate influenced an increase in fatty acis production, which can be used for biodiesel synthesis. However, for this strain, the production of PUFAs was favored, which may lead to a decrease in the quality of biodiesel.

Wensel et al. [31] also analyzed the influence of wheat straw hydrolysate on the cultivation of the microalga *Chlorella vulgaris* ALP2 and observed an increase in lipid production, reaching 0.045 g of triacylglycerols (TAG) L^−1^.day^−1^, revealing high concentrations of palmitic acid (C16:0) and oleic acid (C18:1) in its composition. A lipid profile with an optimal balance of saturated and monounsaturated fatty acids results in the production of high-quality biodiesel, with quality parameters within the values required by international standards.

These results clearly demonstrate the potential of utilizing residual lignocellulosic biomass as a supplement in algae and microalgae cultivation, adding value to the waste material and ensuring increased biomass production and, consequently, higher fatty acid production, which serves as raw material for biodiesel synthesis.

#### 3.5.2. Bioethanol

Biomass is a renewable and promising resource that can be used as a feedstock to produce various types of biofuels, such as bioethanol. However, the use of biomass derived from agricultural residues, such as sugarcane, corn, and rice straw, can increase the production cost of bioethanol due to their high lignin content, which negatively impacts the saccharification process [7,54]. In contrast, photosynthetic biomass represents a promising resource for the production of biofuels and other high-value bioproducts.

The production of bioethanol is also categorized into three consecutive generations based on the biomass used and the respective processing techniques, as shown in Figure 5. 

In recent years, there has been an increase in research focused on using microalgae and cyanobacteria as a source of raw material in biofuel production. Genera such as *Chlorella, Dunaliella*, and *Spirulina* have been found to have a high carbohydrate content, making them important for bioethanol production [54]. For example, several researchers have reported that the genus *Chlorella* exhibits a high concentration of carbohydrates, particularly strains of *C. vulgaris*, with a carbohydrate content ranging from 37% to 55% of its dry weight [7]. Cyanobacteria such as *Anabaena* and *Synechococcus spp*. have also shown high yields (up to 90%) in bioethanol production from consumed sugars [54].

However, the production cost of bioethanol from photosynthetic microorganisms is higher compared to conventional crops due to the high cost of chemicals, culture media, and others [54]. Therefore, the use of lignocellulosic biomass hydrolysates to increase the carbohydrate content of these microorganisms makes the process viable [11]. In a study, researchers compared and evaluated the synthesis of economically important compounds such as carbohydrates, proteins, lutein, and others in the microalga *Chlorella sorokiniana*. The cultures were grown under photoautotrophic conditions using fish farm wastewater and heterotrophic conditions supplemented with spruce hydrolysate. The authors found that the total carbohydrate production in the heterotrophic mode was higher than in the photoautotrophic mode, with values of 8.66% and 6.39%, respectively. This demonstrates an effective source for microalgal carbohydrate production.

Carbohydrates obtained from the cultivation of these microorganisms are commonly derived from starch found in the chloroplasts, as well as cellulose/polysaccharides present in the cell walls [7]. However, for the utilization of these carbohydrates in the fermentation process to obtain bioethanol, the polysaccharides need to be hydrolyzed into fermentable sugars. This is necessary due to their high structural and chemical complexity, which prevents fermentative microorganisms from directly absorbing them from microalgae. Therefore, the pretreatment of this microalgal biomass is critical to reduce the complexity of these chains [59].

The efficiency of the pretreatment is directly related to the morphology of the algae used, specifically the composition of the cell wall [54]. Therefore, microalgal biomass can be hydrolyzed through biological (enzymatic), chemical (alkaline or acid), or physicochemical processes [8]. Generally, the most used methods are chemical and biological. Compared to enzymatic methods, acid hydrolysis of microalgal biomass is faster and more easily utilized, and it is considered low-cost. However, these conditions can lead to greater sugar decomposition and the formation of inhibitors. On the other hand, the enzymatic process is slow and costly, but it is considered a sustainable method [7]. Following biomass hydrolysis and the release of sugars, the alcoholic fermentation process takes place. The most commonly used fermentative microorganism in industrial bioethanol production is the yeast *Saccharomyces cerevisiae* [54].

#### 3.5.3. Biobutanol

Biobutanol obtained from lignocellulosic residues is considered an advanced biofuel due to its properties, such as a high energy content comparable to gasoline and superior to ethanol, low volatility, low corrosivity, and low hygroscopicity. It is seen as a more suitable alternative to fossil fuels and can be synthesized through the petrochemical route or by bacterial fermentation that converts mono- and oligosaccharides into acetone–butanol–ethanol (ABE) [21,60]. In addition to being less corrosive and safer to handle due to its lower vapor pressure compared to other fuels, biobutanol has other applications beyond its use as a biofuel. It can be used as a chemical product in various industries, such as coatings, antibiotics, food, and flavorings [61].

Butanol is a “drop-in” liquid biofuel, which means that through ABE fermentation, it can be blended with petroleum-derived fuels and used in vehicles without any modifications. This makes it highly compatible with the existing infrastructure. Many studies have focused on utilizing microorganisms for the production of the ABE matrix, which can be produced without environmental harm [21].

Cell immobilization has been proposed as an effective way to protect microorganisms from the toxic effects of fermentation. In this regard, Muharja et al. [60] highlighted the use of the cocoa pod husk, a lignocellulosic residue, for biobutanol production. They employed a series of processes that involved the addition of surfactants to improve enzymatic hydrolysis performance and supplementation of L-cysteine as support for fermentation with immobilized cells. The supplementation of L-cysteine, combined with extractive fermentation using immobilized cells, increased the concentrations of butanol and ABE to 20.4 g.L^−1^ and 54.4 g.L^−1^, respectively. Similarly, Linares et al. [61] utilized brewer’s spent grain (BSG), a lignocellulosic material, as a supplement for biobutanol production. Through their experiments, they achieved a yield of 91 kg of butanol per ton of BSG and 138 kg of ABE per ton of BSG.

Based on these results, it is known that transitioning from first-generation to second-generation biofuels by using lignocellulosic residues as an economically viable carbon source for supplementation has been favorable for biobutanol production and can be used for ABE fermentation.

The results obtained by Rezaei et al. [53] using triticale straw as a substrate showed that enzymatic hydrolysis with alkaline pretreatment achieved the highest glucose concentration (32.4 g.L^−1^). The highest butanol production occurred after 72 h of fermentation under alkaline conditions using 1% sodium hydroxide with straw hydrolysate at 180 °C for 60 min, resulting in a concentration of 8.4 g.L^−1^. For ABE production, the results were 13.6, 8.5, and 10.2 g.L^−1^ for alkaline pretreatment (180 °C), diluted acid (140 °C), and autohydrolysis (180 °C), respectively.

Tsai et al. [21] also evaluated the production of biobutanol through the ABE fermentation process using renewable raw materials such as rice straw hydrolysate, sugarcane bagasse, and residual microalgal biomass (after pigment extraction). They obtained a biobutanol titer of 9.1 g.L^−1^ and a yield of 0.1 g biobutanol.g^−1^ glucose for non-hydrolyzed rice straw. On the other hand, for hydrolyzed straw in the absence of yeast extract, the biobutanol titer was 13.8 g.L^−1^, the yield was 0.2 g biobutanol. g-glucose^−1^, and production rate was 0.9 g.L^−1^.h^−1^. In the case of sugarcane bagasse, the biobutanol titer was 8.4 g.L^−1^ of biobutanol, with a yield of 0.1 g biobutanol. g^−1^ glucose. However, for microalgal biomass, acid pretreatment drastically inhibited the fermentation performance. Therefore, non-hydrolyzed microalgae were used for biogas synthesis, resulting in 4.3 g.L^−1^ of biobutanol and a yield of 0.09 g biobutanol/g microalgae in ABE fermentation with a substrate loading of 180 g.L^−1^. These results demonstrate that rice straw is an excellent substrate for ABE fermentation due to its high sugar content, considering that glucose is the main monosaccharide present in lignocellulosic biomass, and is the preferred carbon source in suspended-cell fermentation.

#### 3.5.4. Bio-Oil

There is substantial research on the development of new energy sources as alternatives to fossil fuel-derived materials such as petroleum. One prominent alternative is bio-oil, a promising substitute obtained through thermochemical reactions that convert a portion of biomass into a dense brown liquid composed of a variety of organic compounds. Subsequently, this bio-oil can be further processed to obtain high-quality fuels with a high calorific value due to the presence of oxygenated compounds in its composition and with high added value [6,62].

Biomass sources used for bio-oil production can come from various origins. However, certain characteristics contribute to a product with more desirable properties. The elemental composition is one of the factors that can be decisive for bio-oil production. Biomass with high concentrations of oxygen or nitrogen tends to have reduced bio-oil production. However, the presence of nitrogen improves the quality of the bio-oil [62].

This composition is associated with variations in the composition of bio-oil, as observed by Wang et al. [46] in a pyrolysis study of *Chlorella vulgaris* and peanut shells, where oxygenated compounds were more predominant in the bio-oil derived from peanut shells (concentration of 76.36%) compared to the bio-oil obtained from *C. vulgaris* (49.19%). This difference was attributed to the oxygen-to-carbon ratio of peanut shells (molar ratio of 1.0) in relation to the biomass of *C. vulgaris* (molar ratio of 0.5).

In this context, the use of algae biomass has been promising due to its high concentrations of proteins and nitrogen. Generally, microalgae contain proteins, lipids, and carbohydrates as their main components. In some studies evaluating microalgae for the pyrolysis process, the concentrations of these components were found to reach 62.6% for proteins, 8.7% for lipids, and 19.8% for carbohydrates by weight (%wt) [6,46].

The most reported technique for converting algal biomass into bio-oil is pyrolysis, a thermochemical process that occurs in the absence of oxygen and at high temperatures (400–1200 °C). This technique allows the production of bio-oil as well as other products such as biochar and gases. The key determinant for the type of product obtained from pyrolysis is the temperature at which the process occurs. Conventional pyrolysis, also known as slow pyrolysis, occurs at temperatures below 500 °C and favors the formation of biochar. Flash pyrolysis, also called fast pyrolysis, occurs above 500 °C and yields higher proportions of bio-oil. Pyrolysis above 700 °C primarily produces gases [34,62].

The conversion of biomass into bio-oil occurs through the breakdown of large molecules present in the biomass into smaller molecules. Pyrolysis subjects the biomass to high temperatures, and this energy causes the molecules to vibrate and stretch until their structures break apart [5,46].

**Figure 6:**
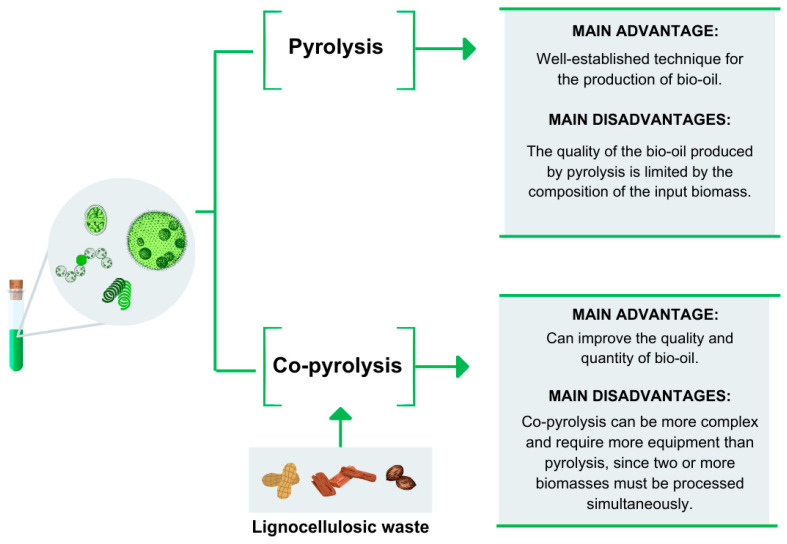
Comparison of the pyrolysis and co-pyrolysis processes.

Despite being a widely used technique, pyrolysis has some disadvantages compared to co-pyrolysis, particularly for bio-oil production (Figure 6). The main drawback is the accumulation of high concentrations of oxygen and water, which are determining factors for fuel quality. Additionally, factors such as pyrolysis temperature, heating rate, and reactor type influence the quality and quantity of the obtained bio-oil [5,62].

For the more efficient production of bio-oil, the co-pyrolysis process is utilized, which yields better results compared to pyrolysis (Figure 6). It involves the simultaneous pyrolysis of two biomass sources, with the aim of utilizing the auxiliary biomass to improve the composition of the bio-oil by adding different compounds than those found in the main biomass. This process also facilitates heat absorption, thereby favoring pyrolysis. Under these conditions, the use of lignocellulosic residues can be advantageous and is being evaluated in the co-pyrolysis process with algal biomass [62,63].

Biomass with a high lignin content tends to form phenolic compounds in bio-oil, while biomass with high nitrogen and protein content results in the presence of nitrogenous compounds in bio-oil. The presence of alcohol has been observed in bio-oil derived from biomass with a high starch content, as indicated by Wang et al. [46]. Regarding production methods, other factors that can hinder the thermochemical reaction include biomass with a high ash content, which tends to form agglomerates that reduce heat and mass transfer in the process. Another factor is a moisture content above 10% (wt), which renders the biomass unsuitable for pyrolysis [62].

The energy efficiency of the evaluated bio-oil is assessed in terms of its heating value. According to Wang et al. [46], the heating value of bio-oil obtained from *C. vulgaris* algal biomass, which represents the energy released when this fuel is completely burned, is 30.4 MJ.kg^−1^ when microwave pyrolysis is used as the method of production. However, this energy value is still lower than that of commercial fuels such as gasoline, which typically ranges from 43 to 45 MJ.Kg^−1^. This indicates the need to improve bio-oil production methods.

In this regard, the combined use of algal biomass and lignocellulosic biomass shows an interesting synergistic effect for bio-oil production. Studies have reported the utilization of lignocellulosic biomass derived from residues such as peanut shells [46], food waste, paper, and wood [63]. In this process, in addition to the production of this biofuel, there is also the possibility of utilizing a waste material, making the production even more sustainable.

Although bio-oil production from algal biomass offers advantages over fossil fuels, such as reduced greenhouse gas emissions and the use of a renewable energy source, there are still technical and economic challenges to be overcome before this technology can be widely adopted on a commercial scale. In this context, the utilization of lignocellulosic biomass has been identified as a promising solution.

### 3.6. Biogas

#### 3.6.1. Biohydrogen

Hydrogen (H_2_) is becoming increasingly promising to produce sustainable biofuels due to its renewable, long-lasting, and cost-effective nature. Its combustion only produces water molecules, making it a promising alternative to fossil fuels [6]. In conjunction with this sustainable characteristic, agricultural and agro-industrial residues have been used for various biotechnological purposes, including biofuel production, due to their lignocellulosic biomass rich in fermentable sugars, primarily composed of three types of polymers: cellulose, hemicellulose, and lignin. The carbohydrates in this biomass undergo a hydrolysis step, converting them into fermentable sugars, thus enabling the conversion of raw materials into bioenergy synthesis [41].

Among the various methods of hydrogen production, the most recent one is the fermentative pathway called dark fermentation, which utilizes lignocellulosic biomass and effluents as raw materials, known as biohydrogen. During this fermentation process, fructose, xylose, strict anaerobes, and oxygen release hydrogen gas. In this regard, Bolonhesi et al. [41] observed that among the agro-industrial residues that can be utilized in dark fermentation, the non-edible parts of cassava, such as the stems, can be used as carbohydrate sources for fermentation, reducing the environmental waste production and adding value to the residues. The improper disposal of many waste materials can cause environmental issues. The group also found that it is possible to immobilize microbial cells on lignocellulosic material to enhance carbohydrate availability, resulting in the presence of sucrose (1.8 and 1.7 g.L^−1^), fructose (1.0 and 0.8 g.L^−1^), and maltose (0.4 and 0.2 g.L^−1^) as the obtained results.

The hydrolysis process has also been proven to be efficient in hydrogen synthesis, achieving the highest hydrogen production rate (VHPR) and hydrogen yield (HY) of 0.7 LH_2_L^−1^d^−1^ (VHPR) and 388 mLH_2_g^−1^ Carb (HY) and 0.7 LH_2_L^−1^d^−1^ (VHPR) and 370 mLH_2_ g^−1^ Carb (HY) for AH and SH assays, respectively. In comparison to the control assay, a 40% decrease in hydrogen production was observed, resulting in a VHPR of 0.4 LH_2_L^−1^d^−1^ and HY of 244 mLH_2_g^−1^ Carb. These results highlight the significance of residual lignocellulosic biomass in biohydrogen production as it increases the availability of fermentable carbohydrates through the hydrolysis process. This not only removes waste from the environment but also adds value to its use in renewable energy generation [41].

#### 3.6.2. Biomethane

Biomethane is a biogas that can be produced from various feedstocks. Recent research has focused on utilizing lignocellulosic residues and combining them with other processes. Rezaei et al. [53] investigated the use of hemicellulosic liquor from triticale straw as a substrate, obtained through three pretreatment methods (acidic, alkaline, and neutral), for anaerobic digestion to produce biomethane in the integration of “cellulosic butanol” and “hemicellulosic biogas” production. The best result for methane production was achieved through neutral self-hydrolysis at 140 °C for 60 min, yielding 430 mL.g^−1^ VSS. In another study by Santos et al. [48], interesting results were obtained for methane production using hydrolysate from coffee husks in both single-stage (methanogenic) and two-stage (acidogenic followed by methanogenic) processes. In the single-stage process, methane production reached 36 NmL CH_4_/g of coffee husks with an energy recovery of 0.064 kJg^−1^. The addition of activated carbon to remove toxic components from the hydrolysate increased the methane production to 86 NmL CH_4_/g of coffee husks, with an energy recovery of 0.5 kJg^−1^. However, in the two-stage process, methane production was 49 NmL CH_4_/g of coffee husks.

In addition to the utilization of lignocellulosic residual biomass, other renewable feedstocks have been investigated as sources for the synthesis of biofuels as their production cycle is less harmful to the environment compared to petroleum-derived fuels [20]. Therefore, microalgae and cyanobacteria biomass, rich in carbohydrates, proteins, and lipids, are emerging as an alternative for biomethane production. However, the anaerobic digestion of microalgae faces challenges in scaling up due to the high costs associated with the collection, cultivation, and refinement processes required for biogas generation [6].

To overcome this obstacle, Rempel et al. [20] proposed a way to reduce the impacts generated by the biofuel production cycle by utilizing residues from the saccharification and fermentation processes of bioethanol production for biomethane synthesis using *Spirulina platensis* biomass. They evaluated three routes for methane synthesis using the biomass of the cyanobacterium Spirulina platensis. The best result was obtained through the direct conversion of cyanobacterial biomass into biomethane, with an energy potential of 16,770 kJ.kg^−1^. Their findings demonstrated the potential of using microalgae and residues as promising alternatives in biofuel production, contributing to the field of renewable energy.

## 4. Conclusions

The need to replace fossil-based raw materials with renewable sources for energy production is evident given the numerous environmental disasters caused by climate change. Algae and microalgae, due to their vast metabolic diversity, hold potential as substitutes for these non-renewable sources in energy production. Several studies have demonstrated the capacity of their biomass to generate high-tech and value-added bioproducts. However, it is still necessary to find the means to make the production of sufficient biomass for large-scale commercialization feasible. Based on the research conducted in this article, it has been reaffirmed that the using residual lignocellulosic biomass is a strong choice for cultivating algae and microalgae. Through supplementation with these biomass sources, it is possible to obtain larger quantities of biomass as well as primary and secondary metabolites. Additionally, this approach enables the removal of lignocellulosic residues from the environment, preventing their accumulation and improper disposal. Moreover, it adds value to the residues by obtaining fractions and metabolites with high market value. The advantages observed when using residual biomass in combination with algae and microalgae cultivation for global energy production are indisputable. These benefits include producing clean biofuels, which reduce environmental pollution, and synthesizing them from renewable raw materials. These renewable resources have the potential to ensure a habitable atmosphere for future generations by mitigating environmental catastrophes and reducing pollutants in the environment.

## Figures and Tables

**Table 1:** Bioproducts derived from algae and microalgae biomass.

Lineage	Compound	Bioproduct	Reference
*Chlorella pyrenoidosa*	PUFAs	Food supplement: Omega-3	[14]
*Phaeodactylum tricornutum*	Fucoxanthin/PUFAs	Nutraceuticals: Anti-obesity, anti-diabetic, and anti-cancer/omega-3	[9]
*Stigeoclonium* sp. B23	Neutral lipids	Bioplastics: Polyhydroxybutyrate (PHB)	[15]
*Nannochloropsis* sp. BR2	Carotenoid	Natural antioxidant: Betacarotene	[16]
*Scenedesmus obliquus* SAG276-10	Fatty acids	Biofuel: Biodiesel	[17]

**Table 2:** Topics used to conduct this study, applied in Scopus, Web of Science, and PubMed.

Topics	Databases
Lignocellulosic waste AND valorization AND hydrolysate AND microalgae	WOS, SCOPUS AND PUBMED
Microalgae AND lignocellulosic waste	
Lignocellulosic waste AND biomass AND hydrolysate AND microalgae	
Microalgae AND biomass AND hydrolysate	
Microalgae AND lignocellulosic waste AND biorefinery	
Microalgae OR algal AND biorefinery AND biomass	
Lignocellulosic waste AND valorization AND hydrolyzed	
Waste biomass AND lignocellulosic AND hydrolyzed AND algae	
Microalgae AND lignocellulosic biomass AND biofuel	
Lignocellulosic AND cyanobacteria	
Lignocellulosic waste AND microalgae AND carbon source	
Carbon source AND lignocellulosic waste AND microalgae	
Biofuel AND residual biomass AND hydrolyzed	
Biomass valorization AND cyanobacteria	
Biomass valorization AND waste AND biorefinery	
Biomass waste AND Amazonic AND valorization	
Feedstock valorization AND agro-industrial wastes AND lignocellulosic waste biomass	
Hemicellulosic AND biofuel AND hydrolysate	
Hemicellulosic AND biofuel AND hydrolysate AND microalgae	
Lignocellulosic AND biohydrogen AND microalgae	
Lignocellulosic AND hydrolyzed AND carbon source	
Lignocellulosic waste AND recovery AND hydrolysate	
Lignocellulosic waste AND Amazonic AND valorization	
Lignocellulosic waste AND hydrolysate AND carbon source	
Lignocellulosic waste AND hydrolysate	
Lignocellulosic waste AND microalgae/cyanobacteria AND hydrolysate	
Microalgae AND biomass AND waste	
Microalgae AND cocoa waste AND biofuel	
Microalgae AND corn waste AND biofuels	
Microalgae AND hydrolyzed AND corn waste	
Microalgae AND hydrolyzed AND waste	
Microalgae AND lignocellulosic biomass AND biofuel	
Microalgae AND lignocellulosic waste AND hydrolysate AND carbon source	
Photosynthetic microorganism AND lignocellulosic waste AND hydrolysate AND carbon source	
Pyrolysis AND microalgae AND bio-oil	
Waste AND husk AND reuse	
Microalgae AND waste AND hydrolysate	
Lignocellulosic waste OR husk AND valorization	
Microalgae AND biomass AND waste	
Lignocellulosic waste AND valorization AND hydrolysate AND microalgae	
Microalgae AND lignocellulosic waste	
Lignocellulosic waste AND biomass AND hydrolysate AND microalgae	

**Table 3:** Contents of the lignocellulosic composition of different agricultural residues.

Substrate	Cellulose (%)	Hemicellulose (%)	Lignin (%)	Reference
Coffee husk	32.5	20.8	27.1	[48]
Açaí seed	8.5	48.1	16.4	[49]
Sugarcane bagasse	45.7	23.4	18.6	[35]
Citrus peel	22.4	8	0.6	[39]
Lentil shell	27.7	8	7.1	[50]
Coconut shell	23.3	12.2	40	[51]
